# Y-chromosome phylogeny in the evolutionary net of chamois (genus *Rupicapra*)

**DOI:** 10.1186/1471-2148-11-272

**Published:** 2011-09-26

**Authors:** Trinidad Pérez, Sabine E Hammer, Jesús Albornoz, Ana Domínguez

**Affiliations:** 1 Departamento de Biología Funcional, Genética, Universidad de Oviedo, Julián Clavería 6, 33006 Oviedo, Spain; 2 Institute of Immunology, Department of Pathobiology, University of Veterinary Medicine Vienna, Veterinaerplatz 1, A-1210 Vienna, Austria

## Abstract

**Background:**

The chamois, distributed over most of the medium to high altitude mountain ranges of southern Eurasia, provides an excellent model for exploring the effects of historical and evolutionary events on diversification. Populations have been grouped into two species, *Rupicapra pyrenaica *from southwestern Europe and *R. rupicapra *from eastern Europe. The study of matrilineal mitochondrial DNA (mtDNA) and biparentally inherited microsatellites showed that the two species are paraphyletic and indicated alternate events of population contraction and dispersal-hybridization in the diversification of chamois. Here we investigate the pattern of variation of the Y-chromosome to obtain information on the patrilineal phylogenetic position of the genus *Rupicapra *and on the male-specific dispersal of chamois across Europe.

**Results:**

We analyzed the Y-chromosome of 87 males covering the distribution range of the *Rupicapra *genus. We sequenced a fragment of the SRY gene promoter and characterized the male specific microsatellites UMN2303 and SRYM18. The SRY promoter sequences of two samples of Barbary sheep (*Ammotragus lervia*) were also determined and compared with the sequences of Bovidae available in the GenBank. Phylogenetic analysis of the alignment showed the clustering of *Rupicapra *with *Capra *and the *Ammotragus *sequence obtained in this study, different from the previously reported sequence of *Ammotragus *which groups with *Ovis*. Within *Rupicapra*, the combined data define 10 Y-chromosome haplotypes forming two haplogroups, which concur with taxonomic classification, instead of the three clades formed for mtDNA and nuclear microsatellites. The variation shows a west-to-east geographical cline of ancestral to derived alleles.

**Conclusions:**

The phylogeny of the SRY-promoter shows an association between *Rupicapra *and *Capra*. The position of *Ammotragus *needs a reinvestigation. The study of ancestral and derived characters in the Y-chromosome suggests that, contrary to the presumed Asian origin, the paternal lineage of chamois originated in the Mediterranean, most probably in the Iberian Peninsula, and dispersed eastwards through serial funding events during the glacial-interglacial cycles of the Quaternary. The diversity of Y-chromosomes in chamois is very low. The differences in patterns of variation among Y-chromosome, mtDNA and biparental microsatellites reflect the evolutionary characteristics of the different markers as well as the effects of sex-biased dispersal and species phylogeography.

## Background

Phylogenetic relationships within and between animal species often depend on the markers studied, as different genes might have different modes of transmission and different histories [1-3]. In addition, hybridization can result in discordant phylogenies between markers. Increasing evidence points to a contribution of reticulate evolution to the speciation process [4-7]. In this context, information on the phylogenies of different markers for closely related species and subspecies is important to the study of processes underlying speciation [8].

**Figure 1:**
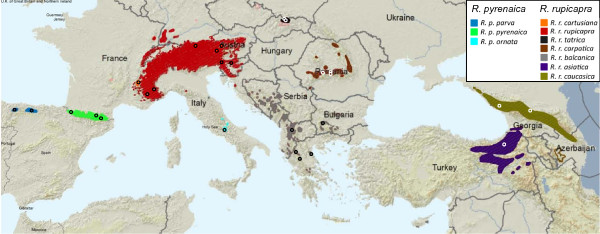
**Geographic distribution of the subspecies of the genus Rupicapra**. Sampling sites are indicated by circles. The map was modified from the distribution map on the IUCN Red List [73].

The study of chamois (*Rupicapra *spp.) allows exploring the effect of historical and evolutionary events on diversification. It is distributed over most of the medium to high altitude mountain ranges of southern Eurasia (Figure 1). At present, chamois populations are classified into two species, *R. pyrenaica *and *R. rupicapra *[9], on the basis of morphological and behavioral characters: *Rupicapra pyrenaica *(with the subspecies *parva*, *pyrenaica *and *ornata*) from southwestern Europe, and *R. rupicapra *(with the subspecies *cartusiana*, *rupicapra*, *tatrica*, *carpatica*, *balcanica*, *asiatica *and *caucasica*) from central and southeastern Europe and western Asia [10]. Analysis of genetic variation in a limited number of subspecies for allozyme loci [11], minisatellites [12], RFLPs of mitochondrial DNA [13] and the major histocompatibility complex [14,15] provided some support for this classification. However, the nominal species are paraphyletic for mtDNA [16,17].

The Quaternary glacial ages probably had a major effect on the phylogeography and evolution of the genus *Rupicapra*, as it did on other animals in Eurasia [18-21]. The Rupicaprini are thought to have originated in Asia during the Miocene period and the sudden appearance of *Rupicapra *fossils in Europe during the middle Pleistocene age has been interpreted as resulting from immigration from the east during a cold climatic phase [21]. In contrast with the fossil record, the divergence between the main mtDNA clades has been estimated around 1.5-3 mya [16,17,22-25] but this cannot be directly assumed to be the divergence time between species. The mitochondrial phylogeny showed three main lineages, originating during the Early Pleistocene [16,17]. Nuclear microsatellite genotypes formed three clearly defined groups as well; however those groups did not exactly match the mitochondrial lineages but are closer to morphology and taxonomic classification. The phylogeographic patterns suggest an evolutionary history with range contractions and expansions related to climatic oscillations during the Quaternary period and reflect a major effect of the Alpine barrier on west-east differentiation. The contrasting phylogenies of mtDNA and nuclear microsatellites for populations of Chartreuse and the western Alps indicated events of range overlap and hybridization among highly divergent lineages in the central area of the distribution. Both markers showed differentiation between all pairs of populations [16,17,26] and a geographic signature in the distribution of variability, suggesting that differentiation occurred without major migrations.

To further elucidate the processes leading to the origin and diversification of *Rupicapra*, we studied the Y-chromosome. The Y-chromosome is paternally inherited and does not undergo recombination at meiosis, providing a marker to study male dispersal [27-29]. The study has the dual purpose of studying the patrilineal phylogenetic position of chamois, compared with *Capra*, *Ovis *and *Ammotragus*, and the male dispersal within the genus *Rupicapra*. We present the analysis of a sequence of a fragment of the SRY gene promoter together with two male-specific microsatellites UMN2303 and SRYM18, in a sample set of 87 males, 40 of *R. pyrenaica *and 47 from *R. rupricapra*, which covers the entire distribution range of chamois. Comparison of the geographic distribution of male-specific markers with mtDNA lineages (defining matrilines) and autosomal markers (biparentally inherited) allows us to follow the evolutionary history of *Rupicapra *in the context of the climatic oscillations of the Pleistocene age.

## Results

### SRY promoter sequences

We have amplified and sequenced 569 nucleotides corresponding to a fragment of the SRY gene promoter from 52 male chamois, 24 of the subspecies *R. pyrenaica *(14 *parva*, 6 *pyrenaica *and 4 *ornata*) and 28 of *R. rupicapra *(5 *cartusiana*, 6 *rupicapraW*, 6 *rupicapra CE*, 3 *tatrica*, 3 *carpatica*, 2 *balcanica*, 1 *asiatica *and 2 *caucasica*). The alignment resulted in only two haplotypes, one in *R. pyrenaica *and the other in *R. rupicapra*. These haplotypes differ only in one nucleotide (site 267 in our alignment), which is A in the haplotype *pyrenaica *and G in *rupicapra*.

**Figure 2:**
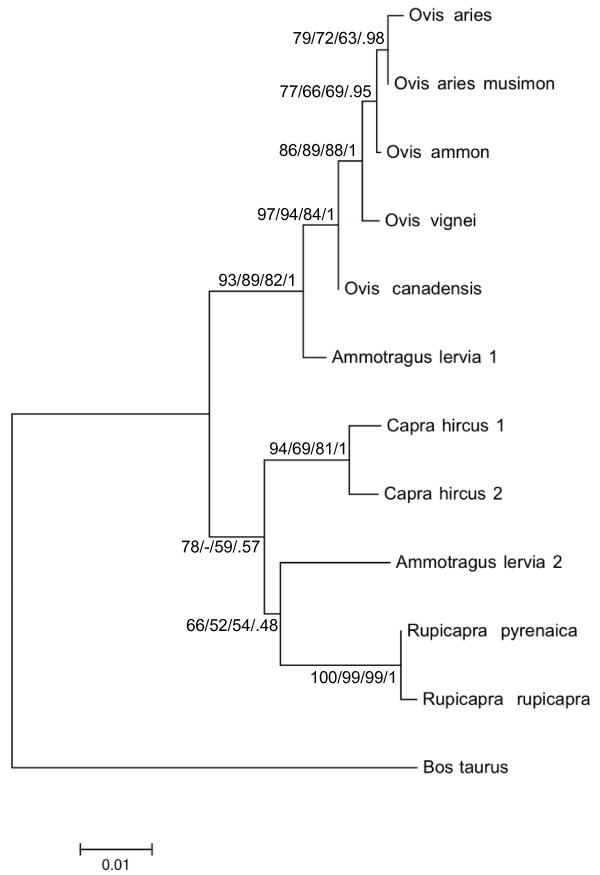
**Phylogenetic tree constructed with SRY-promoter sequences**. Neighbor-Joining tree based on the number of substitutions per nucleotide under the model of Tamura 3-parameter. Numbers at the nodes are bootstrap support using NJ, ML, MP and Bayesian posterior probabilities. *Ammotragus lervia 1 *refers to the sequence of Meadows and Kijas [35] and *Ammotragus lervia 2 *refers to the sequence obtained in this work.

To investigate the evolutionary history of the Y-chromosome of *Rupicapra*, the two haplotypes were aligned with the sequences of other Bovidae available in the GenBank, *Ammotragus lervia*, *Capra hircus*, *Ovis aries *and *Bos taurus *(see Table 1). In addition, two individual *Ammotragus lervia *have been sequenced in our laboratory and both had identical sequence with a deletion of 44 nucleotides with respect to the rest of Bovidae. The aligned dataset contains 531 nucleotides (481 nt, indels excluded) with 78 variable sites of which 34 are fixed and 44 are variable among Caprinae. The phylogenetic relationships were studied using Neighbor-Joining, Maximum Likelihood, Maximum Parsimony, or Bayesian approaches under different models of nucleotide substitution, either the simple model of Jukes-Cantor or the substitution model that describes better the substitution pattern of the dataset, a Tamura 3-parameter model [30] with non-uniformity of evolutionary rate among sites (T92+G). The three parameters were nucleotide frequencies 0.3392 for A and T, 0.1608 for C and G, Ts/Tv ratio: 1.6281 and rate heterogeneity: 0.4762. For the construction of the Bayesian tree, the model of nucleotide substitution was HKI+G (also appropriate to describe the observed substitution pattern since it has the second lowest BIC score obtained with MEGA) and the parameters were obtained by the program BEAST itself. There were 36 parsimony-informative sites. Model-free Parsimony Analysis performed with MEGA led to three equally parsimonious trees with a total length of 87 steps. The different methods of tree construction all led to topologies with two main well supported nodes (Figure 2), one grouping *Ovis *with the published sequences of *Ammotragus *and the other grouping *Rupicapra*, *Capra *and the sequence of *Ammotragus *obtained in this work. The relationships within this second group varied, depending on the method used for tree construction, and they were poorly supported. All the 10 different Bovidae sequences present, like *pyrenaica*, A in site 267 in our alignment, suggesting that this is the ancestral haplotype.

**Table 1:** References of SRY promoter sequences previously available and obtained in this work

Species	GenBank Accession No	Reference
*Bos taurus*	EU581861.1	[74]
*Ovis aries*	EU938044.1	[35]
*Ovis aries musimon*	EU938022.1	[35]
*Ovis ammon*	EU938024.1	[35]
*Ovis canadensis*	EU938032.1	[35]
*Ovis vignei*	EU938028.1	[35]
*Capra hircus 1*	EU581862.1	[74]
*Capra hircus 2*	D82963.1	[75]
*Ammotragus lervia 1*	EU938019.1	[35]
*Ammotragus lervia 2*	JN547784	Present study
*Rupicapra pyrenaica*	JN547785	Present study
*Rupicapra rupicapra*	JN547786	Present study

The estimated divergence time of the two *Rupicapra *haplotypes from the SRY promoter sequence was 655 kya (95% CI: 10-1,611). The mean substitution rate per nucleotide calculated with TRACER from the MCMC samples was 2.09E-3 ± 1.08E-5 per million years.

### Y-chromosome microsatellites

Out of 14 microsatellite markers tested, only UMN2303 and SRYM18 produced male-specific products. Amplification from 87 males, 40 of *R. pyrenaica *and 47 from *R. rupricapra*, revealed two different length fragments for UMN2303 and seven for SRYM18 (Table 2). For each microsatellite, fragments within each length variant were further analyzed by cloning and sequencing, and the architecture was compared with their homologous loci in other Bovidae. The UMN2303 repeated motif was found to be [TTTTG]_n _differing from the repeat [TG]_n _reported in *Bos taurus*. *Rupicapra pyrenaica *presented two alleles, 125 and 130, differing in one repetition, and *R. rupicapra *was monomorphic, with only the 125 allele. The microsatellite SRYM18 lacks the pentanucleotide [TTTTG] and the dinucleotide [TG] motifs common in sheep [31] and instead presents a trinucleotide [TTC]_m _and a mononucleotide [T]_n _motifs. *Rupicapra pyrenaica *haplotypes were [TTC]_m_A[T]_n _and *R. rupicapra *haplotypes were [TTC]_m_[T]_n_, these two structures were reported in *Ammotragus lerviae *(Acc. N° DQ272449) and *Ovis aries *breed Balami (Acc. N° DQ272459.1), respectively. Combinations of variation in number of repeats in both motifs in *R. rupicapra *resulted in homoplasy, where PCR products with the same size had different sequence architecture. The trinucleotide motif, [TTC]_m_, was polymorphic in the species *R. rupicapra *but not in *R. pyrenaica *while the mononucleotide motif was polymorphic in both subspecies (Table 2).

**Table 2:** Haplotypes of the male-specific region of the Y-chromosome of *Rupicapra *(generated through the combination of the sequence of the SRY promoter and microsatellites UMN2303 and SRYM18), and frequencies across subspecies

	Haplotype description						Frequency											
		
	SRY promoter	UMN2303		SRYM18			*R. pyrenaica*			*R. rupicapra*								
					
Haplotype	A/G	Size	Size	[TTC]m	SNP A/T	[T]n	*par*	*pyr*	*orn*	*cat*	*rupW*	*rupC*	*rupE*	*tat*	*cap*	*bal*	*asi*	*cau*
**Y-Rpyr1**	A	130	102	2	A	9	17 (3)	9 (4)										
**Y-Rpyr2**	A	125	102	2	A	9	1 (1)	7 (3)										
**Y-Rpyr3**	A	125	105	2	A	12			6 (1)									
**Y-RrupA1**	G	125	109	3	T	13					1 (1)	6 (1)	1			1 (1)		
**Y-RrupA2**	G	125	110	3	T	14				1 (1)	2 (1)		2 (1)	5 (4)	6 (4)	3 (2)		
**Y-RrupA3**	G	125	111	3	T	15					5 (1)	4 (1)						
**Y-RrupA4**	G	125	112	3	T	16				4 (1)	1 (1)							
**Y-RrupB1**	G	125	111	4	T	12												3 (3)
**Y-RrupB2**	G	125	113	4	T	14											1 (1)	
**Y-RrupC**	G	125	112	5	T	10												1 (1)

The abbreviated name of the subspecies are: *par*, *parva*; *pyr*, *pyrenaica*; *orn*, *ornata*; *cat*, *cartusiana*; *rupW*, *rupicapraW*; *rupC*, *rupicapraC*; *rupE*, *rupicapraE*; *tat*, *tatrica*; *cap*, *carpatica*; *bal*, *balcanica*; *asi*, *asiatica*; and *cau*, *caucasica*. Number of samples sequenced for SRYM18 are given in parenthesis.

### Network of Y haplotypes

Altogether, ten haplotypes could be differentiated in the *Rupicapra *genus (Table 2). Total Y-chromosome haplotype diversity was 0.82 with on average one distinct haplotype over 8.7 individuals (87/10). Three private haplotypes were found in west chamois *R. pyrenaica*, giving a haplotypic diversity of 51.50% and the other seven haplotypes were private of the east chamois *R. rupicapra *with a diversity of 74.69%.

**Figure 3:**
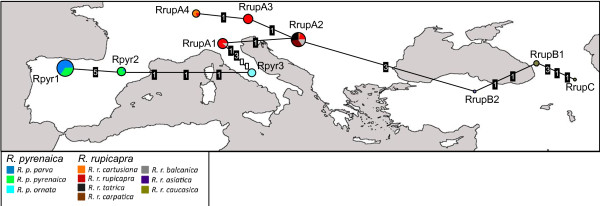
**Network of Y-chromosome haplotypes**. Median-joining network for the Y-chromosome haplotypes constructed using variation at the SRY promoter sequence and at the microsatellites UMN2303 (number of pentanucleotide repeats) and SRYM18 (one SNP, number of trinucleotide repeats and number of mononucleotide repeats). The size of pie areas corresponds to haplotypic frequencies and the proportion accounted for by the different subspecies is represented in different colors as in Figure 1. Different types of mutations in each branch are represented by different symbols (white square: SNP; black squares with a number inside: microsatellites with mononucleotide [1], trinucleotide [3] and pentanucleotide [5] motifs). The network is represented over a map according to the approximate geographical origin of the haplotypes. Branch lengths are not scaled.

The network of haplotypes (Figure 3) revealed two haplogroups that concur with the taxonomy of chamois. These two clades are separated by two nucleotide substitutions (one in the SRY promoter and one in the SRYM18 microsatellite) and a mean distance of 2.11 microsatellite repeats. The connections between haplotypes show a strong geographic signal with links always between neighboring populations. The same network is obtained whether or not the nucleotide substitutions are included.

## Discussion

Analysis of chromosome Y presented a pattern of variation different from the one obtained from either mitochondrial or biparental nuclear DNA, both on diversity as well as on the spread and geographic boundaries of dispersion. However, the east/west phylogeographic signal in the distribution of haplotypes is present again and once more the suture zone places in the Alps. It is remarkable that, contrary to mtDNA and autosomal microsatellites that formed three clades (although not exactly concordant), the variation for the Y-chromosome conforms to the two species currently accepted, *Rupicapra pyrenaica *and *R. rupicapra*. This could explain the concurrence of coat patterns, cranial morphometry and several courtship behavioral patterns in Iberian and Apennine chamois [10,21,32] that remained unexplained from the study of mtDNA and nuclear microsatellites [33].

### Phylogenetic relationships between chamois and other caprini

The phylogeny of SRY promoter shows an association between *Rupicapra, Capra *and the *Ammotragus *sequence obtained in this work (*Ammotragus 2)*. This association concurs with the relationships revealed from the study of the complete mitochondrial genome [34]. Previously, Meadows and Kijas [35] reported a very close relation between the SRY promoter of *Ammotragus *(*Ammotragus lervia 1 *in Table 1 and Figure 2*) *and *Ovis*, but we found 15 differences (3.33%) between this previously reported sequence and the new sequences produced in our laboratory (*Ammotragus lervia 2 *in Table 1 and Figure 2*)*. This large difference is not expected between two individuals of the same species. In contrast, the reported sequence of *Ammotragus *presents only 4 differences (0.83%) with *Ovis canadensis*. Our sequence has been obtained from good quality samples (muscle) from two specimens (both repeated twice) with identical results. So, we think that the sequence reported by Meadows and Kijas could be contaminated with DNA of *Ovis canadensis*. An alternative interpretation to take into account is the polyphyly of *Ammotragus*. The affinities of *Ammotragus *with either *Capra *or *Ovis *have been widely discussed in the literature as it exhibits a particular combination of goat-like and sheep-like characters [36]. The structure of the microsatellite SRYM18 of west chamois is identical to *Ammotragus *and different to most *Ovis*, but the African breed Balami of *O. aries *shares the repeat structure with *Ammotragus *and *Rupicapra*. This observation had lead Meadows and Kijas [31] to hypothesize the possible gene flow from Barbary to domestic sheep. The observed similarities between *Ammotragus lerviae *and the genus *Rupicapra *and the apparent spread of male chamois south to north reopen the question of the possible position of *Ammotragus *as an ancestor of the Caprinae [37]. It can also be noted that *Rupicapra *and *Ammotragus *have similar karyotype with 58 chromosomes [38]. Additional studies of Y-chromosome phylogenies of Caprinae could offer very important information to clarify this issue.

### Patrilineal phylogeography of chamois

**Figure 4:**
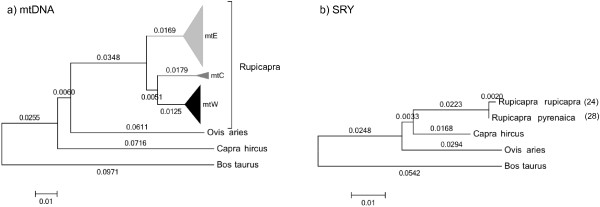
**Comparison of mitochondrial DNA and SRY-promoter phylogenies on chamois**. Neighbor-Joining trees under Jukes-Cantor showing the relationships among chamois and the outgroups *Ovis aries*, *Capra hircus *and *Bos taurus*. a) Tree constructed with a combined sequence of 1708 nucleotides of mtDNA [17]. Sequences in clades mtW, Clade mtC and Clade mtE, in black, grey and white respectively, were collapsed. b) Tree constructed with a sequence of 531 nucleotides of the SRY promoter. For both trees the number of individuals in the external branches is indicated in parentheses.

When comparing the phylogenetic trees based on mtDNA or the sequences of the SRY promoter (Figure 4), a clear difference emerges. All the *Rupicapra *belong to one unique clade for the SRY promoter while three, well differentiated, clades formed for mtDNA. The observed number of substitutions per nucleotide between the pairs of species *Ovis*-*Rupicapra*, *Capra*-*Rupicapra *and *Ovis*-*Capra *for the sequences of mtDNA in our former study [17] were 0.1125, 0.1264 and 0.1186 respectively to be compared with 0.0520, 0.0346 and 0.0489 substitutions per nucleotide respectively for the SRY promoter sequence. The distance between pairs of species for mtDNA is about two or three times that of the SRY promoter, consistent with observations in other mammals including humans [39-42]. The level of differentiation among Y-chromosomes in chamois is remarkably low. The haplogroups Y-Rpyr and Y-Rrup within the *Rupicapra *genus differ by one single nucleotide, leading to an estimated average number of substitutions per nucleotide of 0.0019 that is 24.6 times lower than the average distance between the three clades of mtDNA (0.0468). The time of divergence between the SRY haplotypes estimated from the phylogenetic tree places the split 655 kya, in the middle of the Pleistocene. Thus, all modern chamois seem to descend of one very young male lineage. The low diversity in the number of microsatellite repeats, both between species and within species, compared with the Y-specific evolutionary mutation rate of 2.6 × 10^-4 ^mutations per generation [43], gives further support to this interpretation. Thereafter, our data suggest that the divergence of Y-chromosomal variants took place well after the divergence of mtDNA lineages [17,22,44], in a period compatible with the sudden appearance of *Rupicapra *fossils in Europe [21].

The examination of the Y-chromosome network of haplotypes offers insight into the patrilineal history of dispersion. There is an association between the network of haplotypes and geography (see Figure 3) with a west-east cline of ancestral to derived chromosomes and the signature of the Alpine barrier. Attending to the SNP in the SRY promoter it can be noted that the haplogroup Y-Rpyr presents the ancestral plesiomorph state (A in position 267 in our alignment), shared by all the other species in the phylogeny, while the haplogroup Y-Rrup has the derived state G (transition A > G). Regarding the SNP in the microsatellite SRYM18, Y-Rpyr presents the nucleotide A like *Ammotragus*, hence it could be assumed to represent the ancestral state, while Y-Rrup presents T (transversion A > T). From here we propose that one of the haplotypes of the Y-pyr group represents the ancestral state. The pattern of microsatellite variation within each haplogroup also supports this interpretation as explained below. First, let us recall that the mutation profiles of microsatellites depend on the size of the repeat motif, the rate of mutation of pentanucleotides is about half of that of trinucleotides, and mononucleotide repeats present a very high mutation rate [45]. Our data on pentanucleotide, trinucleotide and mononucleotide difference between *R. pyrenatica *and *R. rupicapra *in number of repeats (0.65, 1.13 and 4.55, respectively) concur with this observation. Hence, microsatellites with longer motifs retain a stronger phylogenetic signal than shorter ones. It has been shown that in microsatellites with few repetitions, mutation is biased towards increase in number of repeats [46,47]. Attending to the network of haplotypes, it can be seen that Rpyr2, sampled from *R. p. pyrenaica *and *R. p. parva*, presents the lowest number of repeats for all, the penta, the tri and the mononucleotide motifs, this is probably the closest to the basal haplotype. The number of repeats of the pentanucleotide microsatellite UMN2303 is variable only in western chamois, the allele 130, with one additional repeat is found in *R. p. pyrenaica *and *R. p. parva*. The number of trinucleotide repeats and mononucleotide repeats of SRYM18 increases west to east. The mononucleotide motif seems to have suffered mutations forth and back in the eastmost populations.

**Figure 5:**
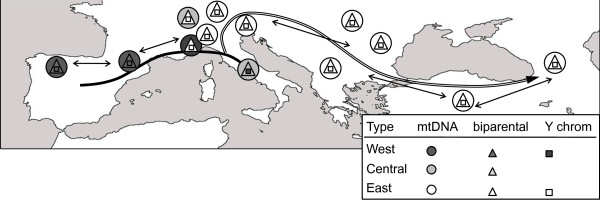
**Summary of geographic distribution of mitochondrial, biparental microsatellites and Y-chromosome variation in chamois**. The affiliation to Clades West, Central and East of extant populations of chamois for the different markers analyzed is represented by forms colored in black, grey and white, respectively. The hypothetical dispersal of male lineages during the Pleistocene is represented by gross lines. Tiny lines represent contraction-expansion of populations within a limited geographical range.

We conclude that the patrilineal dispersion of *Rupicapra *was south-west to north-east. The cline of ancestral to derived chromosomes could be originated by serial funding events, probably related to the glacial-interglacial cycles of the Quaternary. Male dispersion could start from the Iberian Peninsula or from Italy (Figure 5) to the east and the two haplogroups differentiate one to each side of the Alps. The lower number of repeats points to haplotype Rpyr2 present in Iberia as the closest to the ancestor. Alternatively, the haplotype Rpyr3 in the center of the network and differing from Rpyr2 solely in the mononucleotide could be also a good candidate.

### Differences between markers in patterns of variation and the evolutionary history of chamois

The discordant patterns of mtDNA and autosomal microsatellites that have been described in a previous study [17] indicated the occurrence of hybridization among divergent lineages of chamois in the central area of the distribution. Chromosome Y data add complexity to this mosaic; the geographical sorting of variation for the different kind of marker is summarized in Figure 5. The three kinds of markers have specific evolutionary characteristics that need to be taken into account to explain differing variation patterns [27]. These differences are on (1) mutation rates, (2) selection, (3) effective population size and (4) dispersal. (1) Being the Y a male-specific chromosome, and given that the mutation rate is higher in males than in females [48], we would expect higher divergence for Y-chromosome than for autosomal microsatellites; our data are contrary to this expectation. (2) Positive selection could act on the whole Y-chromosome and, given that the Y-chromosome does not recombine, it will be selected as a block in a selective sweep with the consequence of homogenization [28]. In addition, the lack of recombination must be responsible for the effect known as Muller's ratchet, the random accumulation of deleterious mutations that cannot be removed by recombination [49,50]. Selection against these mutations further reduces variability. Selection has been proposed as the main factor to explain low levels of genetic variability in the Y-chromosome [51] and must contribute to the reduced variability among Y-chromosomes in chamois. A similar pattern was found in Ursidae where Y-chromosome genes have fewer substitutions than expected in external branches [41]. (3) The chromosome Y and mtDNA both have effective population size of one quarter of that of an autosome because they are haploid and transmitted by one sex, but the effective population size of Y-chromosome must be lower because the variance in number of descendants is larger in males than in females, especially in a polygynous species. Hence, the Y-chromosome is more prone to genetic drift effects. However, genetic drift cannot be invoked as the main cause for the Y-chromosome phylogeography because in such case no such strong geographical signal would be expected. (4) Differences in male and female dispersal can be related to different patterns of variation for patrilineal, matrilineal and biparental markers. Recent migration of males could be responsible of the low Y-chromosome diversity observed.

Taking into account the above considerations, together with the assumed Asiatic origin of *Rupicapra *and the similarity of the Y-chromosome of *Rupicapra *and *Ammotragus*, we propose two speculative hypotheses to explain the observed results: a) The *Rupicapra *genus has its origin in southern Europe and spread west to east. The differences in divergence between mtDNA and Y-chromosome are due to the particular evolutionary characteristics of the Y-chromosome, or b) males from an ancestral population highly differentiated from a second ancestral taxa present in Europe entered from the south, hybridizing and spreading westwards. Under the first hypothesis, the origin of the genus *Rupicapra *needs to be placed in south Europe from where it spread eastwards. This is contrary to the general belief that chamois, as caprines in general, originated in Asia [21] but, following Ropiquet and Hassanin [23], this hypothesis is not really supported by the fossil record. These authors propose that caprines originated in the Mediterranean islands during the Miocene and from here may have invaded Africa, Europe and Asia after the Tortonian salinity crisis (7.8-7.6 mya) or after the Messinian salinity crisis (6-5.3 mya). The similarity among Y-chromosomes under this hypothesis must be attributed to male dispersal and female phylopatry, together with selective sweeps and purifying selection that acted over the chromosome as a whole. The second hypothesis is the hybridization between two ancestral Caprinae in the beginning of the evolutionary history of the genus *Rupicapra*. The hybridization between highly divergent lineages at speciation of *Rupicapra *had been previously hypothesized after the observation of a pseudogene of cytochrome *b *from a highly divergent lineage in the nucleus of present day chamois [24,37,52]. An ancestral Caprinae related with *Ammotragus *could have reached the south Mediterranean, most probably the Iberian Peninsula, and hybridize with the ancestral *Rupicapra *female. The dispersal of species across the strait of Gibraltar during Pleistocene has been proposed for other vertebrates [40,53,54] and has been related to the substantially lower sea level associated with major Pleistocene glaciations [40]. New male Y-chromosome lineages would have dispersed during the Pleistocene, replacing older ones. Hybridization, after secondary contact among related taxa, has been inferred in the evolution of the genus *Capra *[55] as well as in the evolution of some species of *Bos *[56] and has been recently observed between native and introduced species of *Cervus *in Scotland [57]. It can be added that hybrids from *Ammotragus *male and *Capra *female have been artificially obtained [58]. Many recent studies have shown contrasting phylogenies for different kinds of markers that lead to the view of hybridization as an important mechanism in the evolution of animal species [5,7].

Our results on Y-chromosome reconcile the sudden occurrence of *Rupicapra *in the middle-late Pleistocene with the existence of very old mtDNA lineages. In addition, the proposed migration of chamois west to east can explain the reported more conservative features of *R. pyrenaica *[33]. The alternative hypotheses presented here can be tested in future research that includes the comparative study of chromosome Y in a broader representation of Caprinae and additional nuclear sequences of the different populations of *Rupicapra *to search for the signature of possible hybridizations.

## Conclusions

The Y-chromosome is highly informative to follow the dispersal of populations of Caprinae. The phylogenetic analysis of the SRY gene promoter shows an association between *Rupicapra*, *Capra *and *Ammotragus*, not in agreement with a previous report, which grouped *Ammotragus *with *Ovis*. The structure of the SRYM18 microsatellite of *Rupicapra *is equal to *Ammotragus *and the African breed Balami of *O. aries*. The patrilineal relationships of Caprine deserve a reinvestigation. Within *Rupicapra*, the diversity of Y-chromosomes is very low. The combined data define 10 Y-chromosome haplotypes forming a west-to-east geographical cline of ancestral to derived alleles. Haplotypes form two haplogroups, which concur with taxonomic classification, instead of the three clades formed for mtDNA and biparental microsatellites. We propose that, contrary to the presumed Asian origin, the paternal lineage of chamois originated in the Mediterranean, most probably in the Iberian Peninsula, and dispersed eastwards through serial funding events during the glacial-interglacial cycles of the Quaternary. The differences in patterns of variation among Y-chromosome, mtDNA and biparental microsatellites reflect the evolutionary characteristics of the different markers, as well as the effects of sex-biased dispersal and species phylogeography.

## Methods

### Sampling, DNA extraction and sex determination

Samples of the 10 recognized subspecies of chamois were collected from 1992 until the present, covering the distribution range of the genus *Rupicapra *(see Figure 1) and have previously been analyzed for autosomal microsatellites and mtDNA [16,17,26]. For large populations, where hunting is allowed, samples were either of muscle or skin preserved in 96% ethanol by gamekeepers, or teeth from skulls sent to taxidermists. For protected populations, samples were obtained from animals found dead; tissues, as well as their conservation method, were diverse (hair, bone, salted skin and muscle in ethanol) and were sent by biologists. Two muscle samples of Barbary sheep (*Ammotragus lervia*) one from Sierra Espuña (Murcia, Spain) and the other from Caldera de Taburiente (La Palma, Canary Islands, Spain) were included.

Due to the different origin and type of the material included in this study, different methods of DNA isolation were used. DNA from bones or teeth was extracted by a method modified from Catanneo et al. [59] as described [26]. For soft tissue samples, DNA was extracted either with the phenol/chloroform method [60] using Chelex, following Estoup et al. [61] or using the 'DNeasy Tissue kit' (Qiagen, Hilden, Germany). Finally, 56 of the 215 samples were collected and the DNA was extracted in the laboratory of Vienna (Austria) following the protocol described in the Genetic Analysis Manual (LI-COR, Inc. 1999), followed by a standard phenol/chloroform extraction and DNA precipitation procedure [60].

We determined the sex of each individual sampled using the SE47/SE48 sex identification primers [62]. SE47/SE48 primers amplify one DNA fragment for females (indicating the presence of X-chromosome) and two for males (indicating the presence of a X and a Y chromosome). Reactions were performed in a final volume of 20 μl containing 2 μl (≈ 40-70 ng) DNA, 0.5 mM of each primer, 1x PCR Buffer, 200 mM of each dNTP, 2.5 mM MgCl_2 _and 1 U of Taq DNA polymerase (Qiagen, Hilden, Germany). Amplification was carried out in PE GeneAmp PCR 9700 thermal cycler (Applied Biosystems, Foster City, CA) with an initial step of 5 min at 94°C, 35 cycles of 30 s at 94°C, 30 s at 60°C and 1 min at 72°C, followed by 5 min at 72°C. PCR products were visualized on 2% agarose gels, pre-stained with ethidium bromide. We tested 222 samples, 135 were discarded either because they were from females (83 samples) or they did not amplify (52 samples), and 87 male samples, 40 from *R. pyrenaica *and 47 from *R. rupicapra *were used in the study of Y-chromosome.

### Chromosome Y genotyping

A total of fourteen Y microsatellite loci and six Y-chromosome sequences were tested on chamois for amplification and male specificity (Pérez et al. in preparation). Only two microsatellites, one bovine UMN2303 [63] and one ovine SRYM18 [31] and one male specific sequence, a fragment of the 5'-promoter of the *sex determining region Y *(SRY) gene amplified with primers designed from *Ovis *[31], gave consistent male-specific products and were chosen for further analysis.

The microsatellite loci UMN2303 and SRYM18 were amplified in 20 μl PCR reactions containing 2 μl (≈ 40-70 ng) DNA, 0.5 mM of each primer, 2.5 mM MgCl_2_, 1x PCR Buffer, 200 mM of each dNTP, 2.5 mM MgCl_2 _and 1 U of Taq DNA polymerase (Qiagen, Hilden, Germany). The annealing temperature was 55°C for SRYM18 and 58°C for UMN2303. Fluorescently labeled forward primers were used. Amplification was carried out using the PE GeneAmp PCR 9700 (Applied Biosystems). PCR products were checked in a 2% agarose gel and the product diluted up to 100-fold depending on the signal intensity. One microlitre of the dilution was added to a 12 μl mix of formamide and ROX 400HD (12:0.2) and loaded on an automatic sequencer ABI310 (Applied Biosystems). Microsatellite patterns were examined both visually and using GENESCAN ANALYSIS 3.1 and GENOTYPER 2.5 software (Applied Biosystems).

Sequencing was carried out either cloning the PCR products (UMN2303 and SRYM18 STR loci) or directly (SRY promoter). When cloning was needed, products of amplification were purified using GFX PCR DNA and Gel Band Purification Kit (Amersham Biosciences, Buckinghamshire, UK), and they were directly cloned into the PMOSBlue vector (Amersham Biosciences) and transformed into MOSBlue competent cells according to the supplier's specification. Clones were screened for inserts of the expected size by PCR amplification with the universal primers M13 and T7. For selected clones, plasmid DNA was prepared for sequencing following Sambrock et al. [64]. For direct sequencing PCR-amplified products were purified with the Exo-SAP-IT kit (USB Corporation, Cleveland, OH). Sequencing reactions were performed for both strands using the appropriate primers and the BigDye Terminator v3.1 Cycle Sequencing Kit (Applied Biosystems). Sequencing products were purified with isopropanol precipitation and sequenced in an ABI 310 Genetic Analyzer (Applied Biosystems). The raw sequence data were analyzed using the ABI Prism DNA Sequencer Analysis software v3.4.1. After the occasional observation of differences among the number of mononucleotide repeats in the sequences from SRYM18, the determination of allele size and sequence was repeated for a subset of 16 samples. The results on allele size were always repeatable while the sequences presented errors due to slippage. Hence, the number of mononucleotide repeats was adjusted according to allele size.

### Phylogenetic analysis of SRY promoter sequences

The SRY promoter sequences were manually aligned and edited using MEGA5 [65]. The two different haplotypes identified in *Rupicapra *plus the sequence of *Ammpotragus lerviae *were submitted to NCBI GenBank (accession numbers in Table 1). The phylogenetic relationships of these haplotypes with the available sequences of other Caprinae in the GenBank, *Capra*, *Ovis *and *Ammotragus *were investigated using the sequence of *Bos taurus *as outgroup. Neighbor-Joining (NJ), Maximum Parsimony (MP), Maximum-Likelihood (ML) or Bayesian approaches were used under different models of nucleotide substitution. All positions containing gaps were eliminated. A Neighbor-Joining (NJ) tree of haplotypes based on the simple Jukes-Cantor distance was constructed with MEGA. The reliability of the nodes was assessed by 1,000 bootstrap replicates [66]. Additionally, the optimal substitution model was determined with MEGA 5 using the Bayesian Information Criterion [67]. We used this model to analyze phylogenies both by NJ and Maximum Likelihood (ML) using the Close-Neighbor-Interchange method as tree searching strategy. The topology of the tree was further investigated by model free Maximum Parsimony (MP) as implemented in MEGA, using the Max-mini branch-and-bound algorithm. The MP consensus tree was inferred from 1000 bootstrap replicates. Bayesian analysis was conducted using the Monte Carlo Markov Chains (MCMC) method implemented in BEAST v1.6.1 [68]. A relaxed lognormal model of lineage variation and a coalescent prior with constant size, appropriate when the alignments contain multiple intraspecific sequences, were assumed. The model of nucleotide substitution was HKI+G (also appropriate to describe the observed substitution pattern since it has the second lowest BIC score obtained with MEGA). The empirical nucleotide sequences and a gamma distribution of site heterogeneity with 5 categories of substitution rates were set as priors. Two replicates were run for 25 million generations with tree and parameter sampling every 1,000 generations. Subsequently, the sampling distributions of the two different replicates were combined using LogCombiner and the resulting samples were summarized using the software TreeAnnotator, using a burnin of 2,500 and under the maximum clade credibility option. The percentages of samples recovering any particular clade represent the clade posterior probability. Trees were visualized and edited with FigTree [69].

Divergence time between the two haplotypes of chamois was estimated with BEAST after the analysis of the sequences of *Bos*, *Ovis*, *Capra *and *Rupicapra*. The sequences of *Ammotragus *were not included in this analysis because of the inconsistence of its phylogenetic position that we found in the previous analysis. The conditions of the runs were as before and a relaxed uncorrelated molecular clock was employed. As calibration we used the divergence times of Bovidae (mean 25.8 mya, standard deviation [SD] 0.6 mya), Caprinae (mean 14.1 mya, SD 1.1) and *Capra-Ovis *(11.5 mya, SD 0.9) following Hernández-Fernández and Vrba [70] as a normal distribution prior. We placed monophyly constrains on the group Caprinae and on the groups *Ovis*, *Capra *and *Rupicapra*. The mean rate of nucleotide substitution and its standard deviations were obtained from the Bayesian MCMC sampled values using the program TRACER. The analysis was repeated using the Yule speciation model as prior to check for the effects of the assumed model in the estimate. In addition, the time of divergence was also estimated using only one *Ovis *and one *Capra *sequence (five sequences in total) to check for the effects of having more than one sequence per species on the estimated time of divergence.

### Y microsatellite statistical analyses

We typed 87 individuals (40 of *R. pyrenaica *and 47 of *R. rupicapra*) for the microsatellites UMN2303 and SRYM18 according to size. The SRYM18 microsatellite was found to be compound of two repeated motifs and one SNP. The SNP was treated as a binary trait and the variations in the number of repetitions of the two motifs were treated as two markers. Thus microsatellite individual genotypes were arranged in a matrix of 3 repeated motifs per 87 individuals.

Descriptive statistics analysis was performed with MSAnalyzer v3.12 [71]. We calculated for each repeated motif, in an EXCEL worksheet, the absolute difference in number of repeats (Dad) between the pair of species and its variance to check for differences associated to the length of the repeated motif.

The evolutionary relationships between the haplotypes, of the microsatellite markers alone or the combined haplotypes including binary data (one SNP on the SRY promoter and other SNP in the SRYM18 microsatellite), were analyzed by a Median-Joining network [72] constructed with NETWORK 4.6 (Fluxus Technology Ltd.). This method differs from traditional ones by allowing extant haplotypes to occupy internal nodes. The parameter ε was set to zero (default) to obtain a sparse spanning network. Haplotype components were weighted (w) according to its mutability, increasing weight for the components with lower mutation rate. The weights were as follows: SRYM18 [T]n, w = 1; SRYM18 [TTC]m, w = 2 and UMN2303 [TTTTG]m, w = 3 for the microsatellites. In the network of haplotypes including SNPs as well as microsatellite mutations, the weight for the binary data was set to w = 4.

## Authors' contributions

TP ran the bulk of the laboratory work and data collection and undertook analyses and interpretation. SEH and JA carried out aspects of the molecular lab work and manuscript composition. AD conceived and coordinated the study, analysed SRY promoter sequence and microsatellite data and wrote the paper. All authors read and approved the final manuscript.
